# Using Large Language Models to Assess Burnout Among Health Care Workers in the Context of COVID-19 Vaccine Decisions and Health Beliefs: Retrospective Cohort Study

**DOI:** 10.2196/73672

**Published:** 2025-07-04

**Authors:** Samaneh Omranian, Lu He, AkkeNeel Talsma, Arielle A J Scoglio, Susan McRoy, Janet W Rich-Edwards

**Affiliations:** 1 Brigham and Women’s Hospital and Harvard Medical School, Harvard University, Boston, MA, United States; 2 College of Engineering and Applied Science, University of Wisconsin–Milwaukee, 3200 Cramer Street, Milwaukee, WI, 53211, United States, 1 4142294000; 3 College of Publich Health, University of Wisconsin–Milwaukee, Milwaukee, WI, United States; 4 School of Nursing, University of Wisconsin–Milwaukee, Milwaukee, WI, United States; 5 Department of Natural and Applied Sciences, Bentley University, Waltham, MA, United States; 6 T.H. Chan School of Public Health, Harvard University, Boston, MA, United States

**Keywords:** burnout, health care workers, nurses, COVID-19, health beliefs model, Maslach Burnout Inventory, text classification, LLMs, prompt engineering, LLaMA, fine-tuning, large language models

## Abstract

**Background:**

Burnout among health care workers affects their well-being and decision-making, influencing patient and public health outcomes. Health care workers’ health beliefs and COVID-19 vaccine decisions may affect the risks of burnout. Therefore, understanding the interplay between these crucial factors is essential for identifying at-risk staff, providing targeted support, and addressing workplace challenges to prevent further escalation of burnout-related issues.

**Objective:**

This study examines how burnout is impacted by health beliefs and COVID-19 vaccine decisions among health care workers. Building on our previously developed Health Belief Model (HBM) classifier based on the HBM framework, which explains how individual perceptions of health risks and benefits influence behavior, we focused on key HBM constructs, including the perceived severity of COVID-19, perceived barriers to vaccination, and their relationship to burnout. We aim to leverage natural language processing techniques to automatically identify theoretically grounded burnout symptoms from comments authored by nurses in a large-scale, national survey and assess their associations with vaccine hesitancy and health beliefs.

**Methods:**

We analyzed 1944 open-ended comments written by 1501 vaccine-hesitant nurses, using data from the Nurses’ Health Study surveys. We fine-tuned LLaMA 3, an open-source large language model with few-shot prompts and enhanced performance with structured annotation guidance and reasoning-aware inference. Comments were classified into burnout dimensions—Emotional Exhaustion, Depersonalization, and Inefficacy—based on the Maslach Burnout Inventory framework.

**Results:**

The model achieved a high weighted accuracy of 92% and an *F*_1_-score of 91% for Depersonalization. Emotional Exhaustion was identified in 52% (1003/1944) of comments, correlating strongly with perceived severity (189/323, 59%) and barriers to vaccination (281/650, 43%). Demographic analyses revealed significant variations in burnout prevalence, with older age groups reporting greater burnout.

**Conclusions:**

This study highlights the relationship between burnout and vaccine decision-making among health care workers, uncovering areas for further exploration. By exploring the complex interplay between psychological strain and vaccine hesitancy, this study sets the stage for developing transformative interventions and policies that could redefine workforce resilience and public health strategies.

## Introduction

### Background

The COVID-19 pandemic has profoundly impacted health care workers, intensifying professional burnout and shaping vaccine-related decisions that are critical to public health outcomes [1]. Health care workers faced unprecedented challenges: extreme fatigue, overwhelming stress, and profound grief while managing increased workloads, critical staffing shortages, and limited access to protective equipment [2]. Burnout, defined by the World Health Organization as a syndrome resulting from unmanaged, chronic workplace stress [3], manifests through mental and physical exhaustion, feelings of detachment or cynicism toward one’s job, and a diminished sense of personal accomplishment. These compounding pressures not only affected their well-being but also influenced their perceptions and decisions regarding COVID-19 vaccination, reflecting broader concerns about institutional credibility and policy-driven decisions [4].

Vaccination of health care workers serves a dual purpose: protecting this highly exposed group and preventing iatrogenic spread within health care settings. As one of society’s most trusted professions, health care workers’ attitudes toward vaccination significantly influence public perception and behavior [5]. However, efforts to promote COVID-19 vaccination through community engagement and trust-building initiatives often fell short in addressing health care workers’ fundamental concerns about vaccine mandates, personal autonomy, perceived coercion of incentives, misinformation, and accessibility [6].

Despite substantial research on burnout and vaccine hesitancy, few studies have explored how these factors interact to shape decision-making among health care workers. This gap is especially pronounced in research involving nurses—a population that makes up the backbone of health care systems and has faced disproportionate levels of burnout during the COVID-19 pandemic. Recent studies highlight rising emotional exhaustion and diminished professional fulfillment among nurses, driven by excessive job demands and inadequate institutional support [7,8].

Two theoretical frameworks inform our understanding of the relationship between burnout and vaccine decisions. First, the Total Worker Health Model provides a holistic perspective on how occupational stress, including burnout, influences both personal health decisions and overall job performance [9]. This model evaluates multiple levels of contextual factors, worker characteristics, and organizational elements to assess their impact on both organizational outcomes and worker well-being. Second, the Health Belief Model (HBM) examines how individual perceptions influence health behaviors, including vaccination decisions, through key factors such as perceived susceptibility to disease, perceived severity, perceived benefits of action, and perceived barriers to action [10]. For health care workers experiencing burnout, these constructs may interact in complex ways, reshaping their decision-making processes and potentially exacerbating vaccine hesitancy [11].

Understanding how burnout intersects with vaccine-related health beliefs is critical, yet much of the existing literature relies on structured survey tools that fail to capture the complexity of health care workers’ lived experiences. Free-text narratives provide richer insights into emotional and cognitive processes but pose analytical challenges at scale. To address this, this study applies large language models (LLMs), which excel at interpreting nuanced, unstructured textual data. Unlike traditional machine learning models—which require extensive feature engineering and often miss deeper linguistic or conceptual structures—LLMs can process entire sentences or paragraphs as coherent units, capturing context, tone, and latent psychological meaning [12]. Their capacity to generalize across varied linguistic expressions makes them particularly well suited to analyzing complex constructs such as burnout and vaccine hesitancy in narrative form.

### Related Works

A growing body of research has documented the psychological toll of the COVID-19 pandemic on health care workers, particularly those on the front lines with burnout and vaccine hesitancy emerging as key concerns [13,14]. Burnout—widespread among nurses during the pandemic—has been linked to emotional exhaustion, reduced engagement, and diminished adherence to public health responsibilities [15-17].

Researchers have used a variety of conceptual models to study these effects. Among these, the Maslach Burnout Inventory (MBI) provides a widely recognized framework for measuring and understanding burnout through three key dimensions: Emotional Exhaustion, Depersonalization, and Inefficacy [18]. Previous research has demonstrated that burnout emerged as the most prevalent mental health issue among health care workers during the COVID-19 pandemic, affecting nearly half of the workforce globally and heightening emotional exhaustion, distrust in systems, and impaired decision-making regarding public health measures [19,20].

Galanis et al [21] found that COVID-19-related burnout reduced health care workers’ willingness to be vaccinated, with psychological resilience partially mitigating this effect. Similarly, Limbu et al [22] conducted a systematic review applying the HBM and concluded that perceived barriers, susceptibility, and severity were strong predictors of vaccine hesitancy among health care professionals.

Luna et al [23] applied latent profile analysis to identify distinct burnout patterns in health care workers, linking them to work-related variables such as shift type and job satisfaction, and calling for more personalized burnout interventions. More recently, Nagle et al [24] conducted a scoping review of conceptual burnout models and emphasized the need for integrative, theory-driven tools capable of capturing both individual and systemic drivers of burnout—particularly those that move beyond the limitations of existing instruments like the MBI. Notably, all these studies relied on structured survey instruments and predefined scales rather than open-ended narrative data or language-based classification, which our approach aims to complement.

Despite these advancements, few studies have brought together burnout theory, health behavior models, and LLM-driven text analysis to examine vaccine hesitancy among health care workers. This study addresses that gap by analyzing narrative data from nurses through the dual lenses of MBI and HBM, using LLMs to extract theoretically grounded, fine-grained burnout symptoms from large-scale free-text comments to reveal how burnout and belief structures jointly influence vaccine decisions. The following research questions guided the investigation.

The Main Research Question is: how does burnout correlate with COVID-19 vaccine decisions among health care workers during the pandemic, and how do these correlations vary across demographic factors?

To address this, we break down the main research question into four subquestions:

1. How effective are LLMs in automatically identifying burnout symptoms among health care workers, based on the MBI framework, within the context of the COVID-19 pandemic?
2. What is the relationship between specific MBI burnout dimensions (Emotional Exhaustion, Depersonalization, and Inefficacy) and HBM constructs (barriers, severity, and susceptibility) among vaccine-hesitant (VH) health care workers during the COVID-19 pandemic, and which burnout dimensions are most strongly associated with each HBM construct?
3. How do demographic factors (eg, age, education, and region) influence the relationship between burnout dimensions and vaccine hesitancy?
4. What are the most common themes of the comments in the intersection of burnout dimensions and the HBM?

## Methods

### Ethical Considerations

The study was approved as Protocol 2020P001020 of the Institutional Review Board of Brigham and Women’s Hospital in Boston, Massachusetts, which allowed voluntary survey completion to represent participant consent.

### Data Source, Study Design, and Study Participants

This study used data from our previous investigation of COVID-19 vaccine hesitancy among participants in the Nurses’ Health Study II and Nurses’ Health Study 3, conducted in Winter 2021 [25]. These large-scale, longitudinal cohorts include health care professionals who provided responses on their COVID-19 vaccination intentions and related beliefs. In the prior study, we identified VH individuals based on their responses to the survey question, “Do you plan to receive a COVID-19 vaccine?” Participants who answered “no” or “unsure” (excluding those already vaccinated, intending to get vaccinated, or with missing data) were classified as VH [26]. Of the 4242 participants who provided at least one open-ended comment in the survey (categorized under “vaccine” or “other”), 1501 were classified as VH and included in this analysis. We also considered the personal protective equipment–related comments of these VH participants because these comments are more likely to be work-related and might be a good source for finding work burnout. In total, we analyzed 1,944 open-ended comments provided by VH participants. The vast majority of survey participants (1380/1944,71%) were not actively working at the time of vaccination, while (n=564/1944, 29%) were actively practicing nurses. This cohort, therefore, represents both actively and formerly practicing nurses, highlighting unique perspectives from both groups regarding their vaccination decisions.

In the prior study, these comments were categorized according to constructs of the HBM using supervised learning: perceived barriers, perceived severity, perceived susceptibility, and a category for non-HBM-related comments. This classification enabled us to analyze the underlying health beliefs associated with vaccine hesitancy. This study builds upon this framework, linking these HBM categories with burnout dimensions based on the MBI to explore the intersection of psychological factors and health beliefs in vaccine hesitancy.

For a detailed description of the data collection process and the HBM categorization methodology, refer to our previous work [26].

### Model Selection and Classification Optimization Strategy

We selected LLaMA 3 8B for its open-source accessibility, balanced performance, and ability to run locally, essential for ensuring full control over sensitive health care narratives. The 8B variant offers a practical trade-off between language understanding and computational efficiency, making it suitable for fine-tuning on modest hardware. Its support for Low-Rank Adaptation, a parameter-efficient fine-tuning method, further reduces resource demands by updating only a small subset of model weights [27].

We fine-tuned LLaMA 3 8B on 340 labeled comments, including both original and augmented examples from a manually annotated subset of the dataset. The model was trained using the AdamW 8-bit optimizer, with a linear learning rate scheduler, gradient checkpointing, and mixed-precision (FP16) training. Training was conducted with a small batch size and capped at 60 steps to fit within resource constraints.

To further enhance model understanding of burnout categories, we used the Annotation Guidelines–based Knowledge Augmentation (AGKA) approach developed by Liu et al [28]. AGKA systematically enriches prompts with task-relevant information from annotation guidelines. It has three key components: (1) label definition knowledge, where detailed explanations for each class (eg, Emotional Exhaustion, Depersonalization, and Inefficacy) are retrieved from the annotation guidelines; (2) task instruction formatting, where prompts are carefully structured to include task claims and output expectations; and (3) representative few-shot examples, which demonstrate how to apply the label definitions in context. This design mimics the logic human annotators follow, reading label definitions and using representative cases for guidance. Details of the AGKA prompts are provided in Multimedia Appendix 1.

To improve reliability during inference, we implemented the Reasoning-Aware Self-Consistency (RASC) framework [29]. RASC performs iterative classification on each comment and collects multiple model outputs. When a consistent majority is reached (eg, 70% of outputs align), the system returns that label; otherwise, it continues sampling up to a maximum number of iterations before returning “Undecided.” This approach helps stabilize predictions, particularly for ambiguous or borderline inputs.

### Model Evaluation

In evaluating the model’s performance, we followed a 3-step process. First, we applied the RASC framework, which helped prioritize high-confidence outputs. Then, we measured the model’s performance using metrics of weighted accuracy and *F*_1_-score across the burnout dimensions: Emotional Exhaustion, Depersonalization, and Inefficacy. Finally, we conducted an error analysis to identify recurring misclassification patterns, particularly those involving ambiguous language or overlapping burnout symptoms.

This evaluation framework not only allowed us to assess classification quality but also demonstrated how LLMs can support scalable, interpretable analysis of psychological constructs in free-text narratives. By embedding our approach within the MBI framework and refining it with RASC and AGKA techniques, we provide a replicable methodology for investigating burnout and vaccine hesitancy in health care settings, one that bridges computational and behavioral science in a practical, privacy-preserving way.

### Thematic Analysis

To explore overarching themes at the intersection of burnout and health beliefs, we conducted a one-shot LLM-assisted thematic analysis. Using a prompt-based approach, we provided the base LLaMA 3 model with a single example and instructed it to generate a concise, central theme (limited to 7 words) for each participant’s comment. This method enabled efficient and consistent interpretation of open-ended responses without additional fine-tuning. We then qualitatively reviewed and grouped the model-generated themes to identify recurring patterns related to the intersection of burnout experience and psychological strain.

## Results

### Effectiveness of LLMs in Identifying Burnout Dimension

To evaluate the effectiveness of LLaMA 3 in identifying burnout dimensions based on the MBI framework, we assessed classification accuracy for Emotional Exhaustion, Depersonalization, and Inefficacy using a separate set of 150 unseen labeled comments (50 per dimension). Leveraging the RASC framework with AGKA, the model achieved weighted accuracy ranging from 82% to 92% and *F*_1_-scores from 78% to 91%. The highest weighted accuracy of 92% and an average *F*_1_-score of 91% was achieved for classifying Depersonalization. Emotional Exhaustion and Inefficacy showed slightly lower accuracies of 84% and 82%, respectively. Our classification revealed that Emotional Exhaustion accounted for 52% of the comments, followed by Depersonalization at 25%, and Inefficacy at 11%. This distribution underscores the prominence of Emotional Exhaustion and reflects its strong representation in the dataset.

To evaluate the effectiveness of LLaMA 3 in identifying burnout dimensions based on the MBI framework, we assessed classification performance for Emotional Exhaustion, Depersonalization, and Inefficacy using a separate set of 150 unseen labeled comments (50 per dimension). Leveraging the RASC framework with AGKA, the model achieved weighted accuracy ranging from 82% to 92% and *F*_₁_-scores from 78% to 91%. The highest weighted accuracy of 92% and an *F*_₁_-score of 0.91 were achieved for classifying Depersonalization, which also had a precision of 0.95 and recall of 0.88. Emotional exhaustion showed slightly lower performance, with a precision of 0.81, recall of 0.88, *F*_₁_-score of 0.85, and accuracy of 0.84. Inefficacy had the highest precision (0.99) but a lower recall (0.64), resulting in an *F*_₁_-score of 0.78 and accuracy of 0.82.

### Analysis of Burnout and HBM Construct Intersections

We analyzed the association between specific burnout dimensions and previously categorized HBM constructs: perceived barriers to getting a vaccination, severity of COVID-19, and susceptibility to COVID-19. These associations are represented in Table 1, which illustrates the distribution of burnout dimensions across HBM constructs. Emotional Exhaustion correlated strongly with perceived severity (59%). Additionally, 43% of comments referenced barriers, highlighting both logistical and psychological obstacles to vaccination. Depersonalization was most closely linked to the perceived severity of the disease (27%) and perceived susceptibility to the disease (23%). A smaller proportion of those with depersonalization (17%) referenced perceived barriers, indicating a weaker link to barriers to obtaining the vaccine. Inefficacy showed weaker associations overall, with only 14% of comments reflecting the perceived severity of COVID-19, a total of 8% referenced perceived susceptibility to the disease, and 7% mentioned perceived barriers to getting vaccinated.

**Table 1.** Distribution of HBM^a^ constructs across burnout dimensions^b^.

Burnout dimension	Perceived barriers (%)	Perceived severity (%)	Perceived susceptibility (%)	Non-HBM (%)
Emotional exhaustion	43	59	52	56
Depersonalization	17	27	23	32
Inefficacy	7	14	8	15

a HBM: Health Belief Model.

b Emotional exhaustion shows the highest associations with perceived severity (59%) and non-HBM (56%), while depersonalization and inefficacy show lower percentages across all constructs.

### Demographic Analysis

Further analysis of burnout dimensions by demographic factors revealed significant variations. To examine trends across age groups, we categorized participants into four bins and further assessed the impact of active versus inactive status. As shown in Table 2, Emotional Exhaustion remained consistently high across all groups (≥50%), with the highest prevalence among active nurses younger than 65 years (55%). Interestingly, inactive nurses older than 65 years of age reported similar levels (50%), suggesting that work status may not strongly influence this dimension.

**Table 2.** The prevalence of burnout dimensions among active and inactive nurses, stratified by age group^a^.

Group and burnout dimension	Age (<65 years), n (%)	Age (>65 years), n (%)
Active		
Emotional exhaustion	255 (55)	50 (50)
Depersonalization	136 (29)	21 (21)
Inefficacy	74 (16)	6 (6)
Inactive		
Emotional exhaustion	241 (51)	457 (50)
Depersonalization	112 (24)	211 (23)
Inefficacy	52 (11)	89 (10)

a Emotional Exhaustion remains high across all groups, while Depersonalization and Inefficacy show notable differences, with active nurses younger than 65 years of age reporting the highest levels.

In contrast, Depersonalization and Inefficacy showed more variation. Active nurses, particularly those younger than 65 years, exhibited significantly higher rates of both compared to their inactive counterparts (29% vs 24% for Depersonalization; 16% vs 11% for Inefficacy). Chi-square tests confirmed statistically significant differences across burnout dimensions (*P*<.001), particularly for Depersonalization and Inefficacy across age and activity levels. These findings highlight the distinct stressors faced by active nurses and underscore the need for targeted interventions.

**Figure 1.**
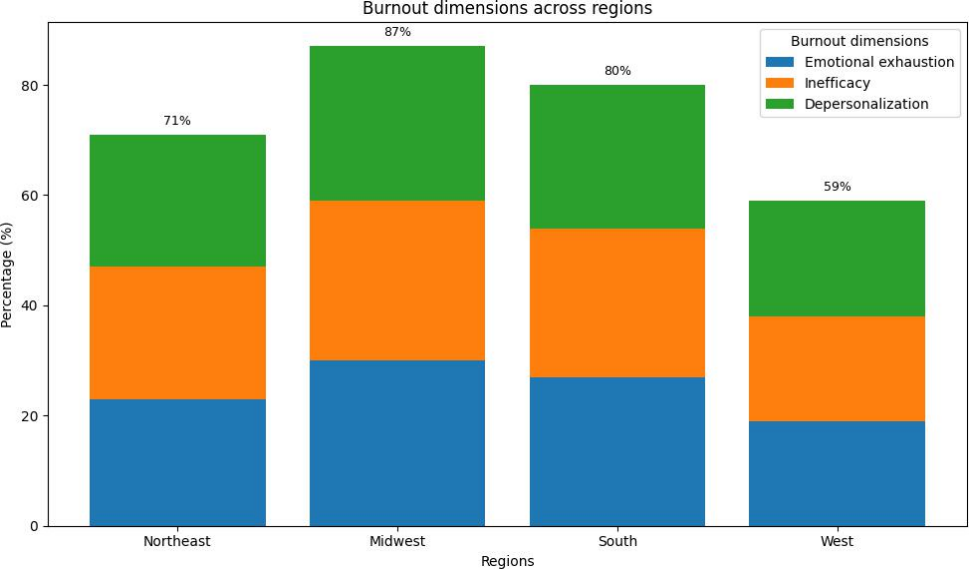
Burnout dimensions across regions. Midwest shows the highest burnout (87%), followed by south (80%) and west (71%).

Regional differences in burnout prevalence were also observed (Figure 1). The Midwest reported the highest total burnout percentage (87%), with Emotional Exhaustion, Inefficacy, and Depersonalization contributing 30%, 29%, and 28%, respectively. The South followed with a total burnout percentage of 80%, while the Northeast region reported a similar pattern at 71%. The West exhibited a lower total burnout percentage of 59%.

### Key Themes at the Intersection of Burnout and HBM Constructs

To address subquestion 4, our thematic analysis identified overlapping concerns at the intersection of burnout dimensions and HBM constructs. Health and safety concerns, including vaccine side effects, efficacy, and misinformation, were dominant among comments highlighting perceived barriers to vaccination. Emotional and psychological strain, characterized by stress, loss, and work-life challenges, further underscored the mental toll of burnout in shaping vaccine hesitancy. In some cases, participants cited low perceived risk due to high COVID-19 recovery rates, which reduced the urgency for vaccination.

Broader themes reflected distrust toward government and political systems, frustrations with systemic obstacles, and the pandemic’s impact on social and family dynamics. Faith and resilience were also significant factors, with many participants relying on spiritual beliefs and community support to navigate vaccine-related concerns. These themes illustrate the complex interplay between burnout dimensions and health beliefs, shaping health care workers’ vaccine decisions.

### Error Analysis

We conducted both quantitative and qualitative error analysis, focusing on predicted versus true labels, classification confidence, and label-specific performance metrics such as precision, recall, and *F*_1_-score.

Initial results revealed key challenges, with multiclass classification achieving only 55% accuracy. Misclassified outputs showed ambiguity and overlapping language, particularly between Emotional Exhaustion and Depersonalization, which frequently co-occurred and shared similar linguistic cues.

For example, comments such as “Changing expectations & policies at work are frustrating causing burnout. Lack of free speech/generally one-way group think causes burnout feeling. Everyone is tired of the isolation and changes to our social lives” could be classified as either Emotional Exhaustion or Depersonalization, depending on interpretation. Phrases like “frustration causing burnout” and “everyone is tired of the isolation” suggest fatigue and feeling emotionally overwhelmed, which align with Emotional Exhaustion. On the other hand, mentions of “lack of free speech” and “one-way group think” could reflect feelings of disconnection or detachment from the work environment, or an insensitive attitude toward colleagues and tasks, aligning more with Depersonalization.

Similarly, the comment “I was working as an R.N. in the pandemic and eventually developed severe anxiety, panic attacks, and depression and am currently on a leave of absence from work for these reasons” strongly reflects Emotional Exhaustion, characterized by emotional and psychological depletion. However, the mention of a leave of absence might also suggest withdrawal, potentially overlapping with Depersonalization and contributing to misclassification.

Depersonalization, in particular, showed high precision but low recall. This discrepancy likely stems from the smaller proportion of comments classified under Depersonalization, leading to more cautious predictions for this dimension and reduced sensitivity to identifying all relevant instances.

## Discussion

### Overview

This study examined the role of burnout and health beliefs in shaping COVID-19 vaccine decisions among health care workers, offering novel insights through the integration of LLM-based text analysis. By leveraging LLaMA 3 for categorizing burnout dimensions and analyzing their intersection with HBM constructs, the findings align with and extend prior research on the psychological and behavioral factors influencing vaccine hesitancy.

### Key Findings and Comparisons

The predominance of Emotional Exhaustion (52% of comments) and its strong correlation with perceived severity (59%) and barriers (43%) underscores the critical role of psychological strain in vaccine hesitancy. These results are consistent with previous studies that link high levels of emotional distress among health care workers with increased concerns about vaccine safety and efficacy [30-33]. Depersonalization, which showed associations with perceived severity (27%) and susceptibility (23%), highlights the psychological detachment experienced by some health care workers, reflecting findings by Zhang et al [34], who identified significant links between burnout dimensions such as depersonalization and factors influencing job satisfaction and turnover intentions among nurses in high-stress settings. In contrast, inefficacy, while less prevalent, aligns with research indicating that feelings of low personal accomplishment may undermine self-efficacy and motivation, thereby dampening proactive health behaviors [35].

Our findings indicate widespread emotional exhaustion across all groups, with at least 50% of both active and inactive nurses reporting high burnout. While we expected active nurses to experience greater burnout due to workplace stress during COVID-19, inactive nurses older than 65 years of age exhibited similar levels, suggesting additional contributing factors. Notably, most inactive nurses in this study were retired, and some reported caregiving responsibilities for spouses, older parents, or grandchildren during pandemic-related closures. These stressors, along with the broader emotional toll of the pandemic, may explain their high burnout levels. Given that data collection occurred early in the pandemic and before mandatory vaccination, we cannot conclude that burnout led to early retirement. However, for those nearing retirement (55-65 years of age), pandemic-related stress may have influenced workforce decisions. The consistently high emotional exhaustion across groups underscores the long-term psychological burden of health care work, warranting further research into postretirement burnout and its lasting effects.

The interplay between burnout dimensions and vaccine hesitancy highlights significant psychological and contextual barriers to vaccination among health care workers. These findings align with prior research, which demonstrates that emotional exhaustion and distrust in systemic structures exacerbate vaccine hesitancy during crises [36,37]. Addressing such multifaceted concerns requires holistic approaches that consider not only health beliefs but also the broader sociopolitical and personal challenges faced by health care workers.

The application of LLaMA 3 with the RASC framework demonstrated robust performance in classifying burnout dimensions, achieving high accuracy and *F*_1_-scores, particularly for Emotional Exhaustion and Inefficacy. These findings underscore the potential of leveraging domain-specific knowledge and few-shot learning strategies for complex classification tasks, aligning with prior research emphasizing the role of targeted annotation and reasoning-based approaches to improve natural language processing model outcomes [28,29]. Future studies could explore fine-tuning strategies to address challenges with overlapping burnout dimensions.

### Policy Implications and Interventions

The findings of this study also have significant implications for policy makers. Systemic interventions are needed to address organizational stressors contributing to burnout, including manageable working hours, adequate staffing ratios, and improved access to mental health resources tailored to health care workers. Transparent communication and inclusive decision-making processes are essential to rebuilding trust in health care systems and alleviating systemic distrust that influences vaccine hesitancy.

Policy makers should also support the development of monitoring frameworks that use real-time data to identify at-risk health care workers. Machine learning–driven tools, such as those leveraging LLMs, could enhance early detection of burnout symptoms through employee feedback systems. Finally, interventions must be culturally sensitive and address the diverse needs of underrepresented health care worker populations. These systemic approaches, informed by this study, could improve workforce well-being and public health outcomes by addressing burnout and vaccine hesitancy at their root causes.

### Limitations and Future Directions

While this study provides valuable insights, several limitations warrant attention. The reliance on self-reported comments may introduce reporting bias, as participants may underreport or selectively emphasize certain factors influencing their vaccine hesitancy. Additionally, the study’s dataset, derived from the Nurses’ Health Study cohorts, includes only female registered nurses, with the majority identifying as White. This demographic homogeneity limits the generalizability of the findings to other health care professions, genders, and ethnic groups. Furthermore, challenges remain in distinguishing overlapping burnout dimensions which require further refinement in annotation guidelines and modeling strategies.

### Conclusions

Future research should explore the longitudinal effects of burnout on vaccine-related behaviors and incorporate diverse health care worker populations. Efforts should also focus on identifying additional burnout factors not included in the MBI. Additionally, designing a preventive framework or tool to monitor and follow up with health care workers for early detection of burnout symptoms could transform interventions. Such tools, integrating real-time machine learning algorithms, could provide tailored interventions to address burnout symptoms before escalation. Expanding the application of LLMs to include multitask learning and fine-tuning with larger annotated datasets may enhance the understanding of complex psychological constructs and their implications for vaccine decisions.

## Supplementary material

10.2196/73672 Multimedia Appendix 1 Annotation Guidelines-based Knowledge Augmentation (AGKA prompts) with emotional exhaustion prompt.

## Abbreviations

AGKA: Annotation Guidelines–based Knowledge Augmentation
HBM: Health Belief Model
LLM: large language model
MBI: Maslach Burnout Inventory
RASC: Reasoning-Aware Self-Consistency
VH: vaccine hesitant
