# Supplementary material for: Using Large Language Models to Assess Burnout Among Health Care Workers in the Context of COVID-19 Vaccine Decisions and Health Beliefs: Retrospective Cohort Study
Source: JMIR Nurs. 2025 Jul 4;8:e73672. doi: 10.2196/73672 (PMC12248134; doi:10.2196/73672)
Supplement: Multimedia Appendix 1 [file nursing-v8-e73672-s001.docx]

## Annotation Guidelines-based Knowledge Augmentation (AGKA prompts) with emotional exhaustion prompt = """

## ### Instruction:

## Determine if the text below indicates WORK-RELATED EMOTIONAL EXHAUSTION based on the Maslach Burnout Inventory (MBI). Emotional exhaustion refers to feeling mentally and physically drained due to chronic workplace stress, marked by fatigue, frustration, and a sense of being overwhelmed, especially when demands consistently exceed emotional resources.

## Based on the definition and examples if the comment reflects WORK-RELATED EMOTIONAL EXHAUSTION, respond with "1"; otherwise, respond with "0".

## ### Examples:

## - Comment: "I have been overworking due to staff shortages and am concerned that this will compromise my health. My sleep hours and quality have been fair, not great."

## Response: 1

## - Comment: "The constant demands of my job leave me feeling drained and unable to find time for myself. It's getting harder to stay motivated."

## Response: 1

## - Comment: "I'm looking forward to the weekend to relax and recharge after a long week at work."

## Response: 0

## - Comment: "Lately, I feel like I can't keep up with the workload, and it's affecting my energy levels."

## Response: 1

## - Comment: "Despite the busy schedule, I still find joy in my daily tasks and interactions."

## Response: 0

## - Comment: "I often feel exhausted after my shifts and struggle to engage with my family."

## Response: 1

## - Comment: "Managing my time effectively allows me to maintain a healthy work-life balance."

## Response: 0

## - Comment: "The relentless pace of work has left me feeling perpetually tired and overwhelmed."

## Response: 1

- Comment: "I feel energetic and passionate about my role, even during peak times."

Response: 0

- Comment: "Chronic stress from work has made it difficult for me to recover during my days off."

Response: 1

## ### Input:

## Comment: {comment}

## ### Response:

## {response}

## """

inefficacy prompt = """

### Instruction:

Determine if the following nurse's comment reflects WORK-RELATED REDUCED PERSONAL ACCOMPLISHMENT based on the definition provided. Respond only with "1" for Yes or "0" for No. Do not include any explanations or additional text.

### Definition:

A nurse is experiencing WORK-RELATED REDUCED PERSONAL ACCOMPLISHMENT if they express:

- Feelings of ineffectiveness or unproductiveness at work.

- A sense of inadequacy or failure in their role.

- Lack of achievement or doubts about their contributions.

### Examples:

- Comment: "I feel like no matter how much effort I put in, nothing I do makes a difference for my patients or my team."

Response: "1"

- Comment: "I am constantly doubting my abilities and feel like I’m falling short of everyone’s expectations at work."

Response: "1"

- Comment: "No matter what I do, I just don't feel like I'm making a meaningful impact."

Response: "1"

- Comment: "I often think that my skills aren't sufficient for the demands of my job."

Response: "1"

- Comment: "There are challenges at work, but I feel like I’m growing and learning every day."

Response: "0"

- Comment: "Sometimes I feel frustrated, but I know my work contributes to the bigger picture."

Response: "0"

- Comment: "I face difficulties at work, but overcoming them makes me feel accomplished."

Response: "0"

- Comment: "Even on tough days, I believe I'm effective in my role."

Response: "0"

### Input:

Comment: {comment}

### Response: {response}

"""

depersonalization prompt = """

### Instruction:

Determine if the text below indicates WORK-RELATED DEPERSONALIZATION based on the Maslach Burnout Inventory (MBI). Depersonalization refers to a sense of emotional distance or detachment from one’s work, colleagues, or clients. It is often marked by cynical, impersonal, or indifferent attitudes, where individuals may treat others more like objects than human beings, reflecting a lack of empathy or engagement.

Based on the definition and examples, if the comment reflects WORK-RELATED DEPERSONALIZATION, ONLY respond "1"; otherwise, respond with "0".

### Examples:

- Comment: "I don’t have the energy to care about patients anymore. It’s just about getting through the day, not making a connection."

Response: 1

- Comment: "I feel disconnected from my colleagues. I try to avoid interactions as much as possible because I don’t see the point."

Response: 1

- Comment: "Sometimes I joke around to lighten the mood, even if it seems a bit cynical."

Response: 0

- Comment: "I feel tired but still care deeply about providing the best for my patients."

Response: 0

- Comment: "Interacting with patients feels like a chore now; I just want to get through my shifts."

Response: 1

- Comment: "I maintain a professional demeanor, even when I'm feeling stressed."

Response: 0

- Comment: "I've started to view my colleagues as obstacles rather than team members."

Response: 1

- Comment: "Despite the workload, I still find joy in helping my patients."

Response: 0

### Input:

Comment: {comment}

### Response:

{response}

"""
